# Circulating adipokines data associated with insulin secretagogue use in breast cancer patients

**DOI:** 10.1016/j.dib.2016.11.060

**Published:** 2016-11-22

**Authors:** Zachary A.P. Wintrob, Jeffrey P. Hammel, George K. Nimako, Zahra S. Fayazi, Dan P. Gaile, Alan Forrest, Alice C. Ceacareanu

**Affiliations:** a State University of New York at Buffalo, Department of Pharmacy Practice, NYS Center of Excellence in Bioinformatics and Life Sciences, 701 Ellicott Street, Buffalo, NY 14203, United States; b Cleveland Clinic, Department of Biostatistics and Epidemiology, 9500 Euclid Ave., Cleveland, OH 44195, United States; c State University of New York at Buffalo, Department of Biostatistics, 718 Kimball Tower, Buffalo, NY 14214, United States; d The UNC Eshelman School of Pharmacy, Division of Pharmacotherapy and Experimental Therapeutics, Campus Box 7569, Chapel Hill, NC 27599, United States; e Roswell Park Cancer Institute, Department of Pharmacy Services, Elm & Carlton Streets, Buffalo, NY 14263, United States

**Keywords:** Adipokine, Insulin, Secretagogue, Breast cancer, Diabetes, Cancer outcomes, Cancer prognosis

## Abstract

Oral drugs stimulating endogenous insulin production (insulin secretagogues) may have detrimental effects on breast cancer outcomes. The data presented shows the relationship between pre-existing insulin secretagogues use, adipokine profiles at the time of breast cancer (BC) diagnosis and subsequent cancer outcomes in women diagnosed with BC and type 2 diabetes mellitus (T2DM). The Pearson correlation analysis evaluating the relationship between adipokines stratified by T2DM pharmacotherapy and controls is also provided. This information is the extension of the data presented and discussed in “*Insulin use, adipokine profiles and breast cancer prognosis*” (Wintrob et al., in press) [1].

**Specifications Table**

**Table:** 

Subject area	Clinical and Translational Research
More specific subject area	Biomarker Research, Cancer Epidemiology
Type of data	Tables
How data was acquired	Tumor registry query was followed by vital status ascertainment, and medical records review
	Luminex®- or enzyme-linked immunosorbent assay- based quantitation of adipokines (adiponectin, leptin, C-reactive protein, interleukine-6, interleukine-1β, interleukine-1Ra, tumor necrosis factor-α, and C-peptide) from plasma samples was conducted.
	A Luminex®200^TM^ instrument with Xponent 3.1 software was used to acquire all data except for C-reactive protein determinations which have been done using a Synergy 2 BioTek multi-mode reader
Data format	Analyzed
Experimental factors	Adipokines were determined from the corresponding plasma samples collected at the time of breast cancer diagnosis
Experimental features	The dataset included 97 adult females with diabetes mellitus and newly diagnosed breast cancer (cases) and 194 matched controls (breast cancer only). Clinical and treatment history were evaluated in relationship with cancer outcomes and adipokine profiles. A biomarker correlation analysis was also performed.
Data source location	United States, Buffalo, NY - 42° 53′ 50.3592″N; 78° 52′ 2.658″W
Data accessibility	The data is with this article

**Value of the data**

• Presented data shows the relationship between pre-existing insulin secretagogues use, adipokine production at the time of cancer diagnosis and breast cancer outcomes.
• This data serves as a benchmark for future investigations targeting pharmacotherapy-induced adipokine modulation in breast cancer.
• The data described here can assist study design of further biomarker evaluation in relationship with the safety and effectiveness of diabetes pharmacotherapy.

## 1 Data

Reported data represents the observed association between insulin secretagogues׳ utilization and the adipokine profiles at the time of breast cancer diagnosis in women with diabetes mellitus (Table 1). Data in Table 2 includes the observed correlations between adipokines stratified by type 2 diabetes mellitus pharmacotherapy and controls.

## 2 Experimental design, materials and methods

Evaluation of adipokine profile association with insulin secretagogue use and BC outcomes was carried out under two protocols approved by both Roswell Park Cancer Institute (EDR154409 and NHR009010) and the State University of New York at Buffalo (PHP0840409E). Demographic and clinical patient information was linked with cancer outcomes and adipokine profiles of corresponding plasma specimen harvested at BC diagnosis and banked in the Roswell Park Cancer Institute Data Bank and Bio-Repository.

### 2.1 Study population

As described in the original research article by Wintrob et al. [1], all incident breast cancer cases diagnosed at Roswell Park Cancer Institute (01/01/2003-12/31/2009) were considered for inclusion (*n*=2194). Medical and pharmacotherapy history were used to determine the baseline presence of diabetes.

### 2.2 Inclusion and exclusion criteria

Inclusion criteria were as follows: minimum 18 years of age at diagnosis, presence of pre-existing diabetes at breast cancer diagnosis, and having available banked treatment-naïve plasma specimens in the Institute׳s Data Bank and Bio-Repository. That is, the blood had to be collected prior to the initiation of any cancer-related therapy (surgery, radiation or pharmacotherapy).

Subjects were excluded if they were male, had prior cancer history or unclear date of diagnosis, incomplete clinical records, type 1 or unclear diabetes status. For a specific breakdown of excluded subjects, please see the original research article by Wintrob et al. [1].

A total of 97 female subjects with breast cancer and baseline diabetes mellitus were eligible for inclusion in this analysis.

### 2.3 Control-matching approach

Each of the 97 adult female subjects with breast cancer and diabetes mellitus (defined as “cases”) was matched with two other female subjects diagnosed with breast cancer, but without baseline diabetes mellitus (defined as “controls”). The following matching criteria were used: age at diagnosis, body mass index category, ethnicity, menopausal status and tumor stage (as per the American Joint Committee on Cancer). Some matching limitations applied [1].

### 2.4 Demographic and clinical data collection

Clinical and treatment history was documented by medical chart review. Vital status was obtained from the Institute׳s Tumor Registry, a local database updated biannually with data obtained from the National Comprehensive Cancer Networks׳ Oncology Outcomes Database. Outcomes of interest were breast cancer recurrence and/or death. For additional details concerning data collection, specific definitions regarding censoring and drug use, and a comprehensive demographic report, please see the original article [1].

### 2.5 Plasma specimen storage and retrieval

All the plasma specimens retrieved from long-term storage were individually aliquoted in color coded vials labeled with unique, subject specific barcodes. Overall duration of freezing time was accounted for all matched controls ensuring that the case and matched control specimens had similar overall storage conditions. Only two instances of freeze-thaw were allowed between biobank retrieval and biomarker analyses: aliquoting procedure step and actual assay.

### 2.6 Enzyme-linked immunosorbent assay and Luminex® assays

A total of 7 biomarkers (adiponectin, leptin, C-reactive protein, interleukine-6, interleukine-1β, interleukine-1Ra, tumor necrosis factor-α, and C-peptide) were quantified using either enzyme-linked immunosorbent or Luminex® assays, as described by Wintrob et al. [1]. A quantitative colorimetric enzyme-linked immunosorbent assay was performed for detection of C-reactive protein, according to manufacturer protocol (Genway Biotek Inc., San Diego, CA). The following Luminex® biomarker panels were utilized in this study: human cytokine/chemokine panel I (MPXHCYTO-60K for interleukine-1β and interleukine-1Ra), human high sensitivity cytokine/chemokine panel (HSCYTO-60SK for interleukine-6 and tumor necrosis factorα), human cardiovascular disease panel I (HCVD1-67AK for adiponectin), and human endocrine panel (HENDO-65K for leptin and c-peptide) produced by Millipore Corporation, Billerica, MA.

### 2.7 Biomarker-pharmacotherapy association analysis

Biomarker cut-point optimization was performed for each analyzed biomarker. Biomarker levels constituted the continuous independent variable that was subdivided into two groups that optimized the log rank test among all possible cut-point selections yielding a minimum of 10 patients in any resulting group. Quartiles were also constructed. The resultant biomarker categories were then tested for association with type 2 diabetes mellitus therapy and controls by Fisher׳s exact test. The continuous biomarker levels were also tested for association with diabetes therapy and controls across groups by the Kruskall-Wallis test and pairwise by the Wilcoxon rank sum. Multivariate adjustments were performed accounting for age, tumor stage, body mass index, estrogen receptor status, and cumulative comorbidity. The biomarker analysis was performed using R Version 2.15.3. Please see the original article for an illustration of the analysis workflow [1].

Correlations between biomarkers stratified by type 2 diabetes mellitus pharmacotherapy and controls were assessed by the Pearson method. Correlation models were constructed both with and without adjustment for age, body mass index, and the combined comorbidity index. Correlation analyses were performed using SAS Version 9.4.

## Funding sources

This research was funded by the following Grant awards: Wadsworth Foundation Peter Rowley Breast Cancer Grant awarded to A.C.C. (UB Grant Number 55705, Contract CO26588), the New York State Council of Health-system Pharmacists Research and Education Foundation (NYSCHP-REF) Oncology Leadership Grant awarded to A.C.C. (UB Grant Number 50151), and the NYSCHP-REF Clinical Pharmacy Award TO A.C.C. (UB Grant Number 53967).

## Figures and Tables

**Table 1:** Adipokines associations with insulin secretagogue use.

Biomarker	Biomarker Grouping	Concentration	Control	No Secretagogue	Any Secretagogue	Unadjusted *p*-value (MVP)			
						*p*^1^	*p*^2^	*p*^3^	Global test
Adiponectin(ng/ml)	Median (25–75th)	–	14.9 (10.7–22.6)	11.3 (6.89–20.9)	11.7 (8.10–17.6)	<0.015 (0.022)	0.008 (0.210)	0.810 (0.770)	0.005 (0.046)
	Quartiles	1.79–8.90	38 (19.6%)	17 (36.2%)	18 (36.0%)	0.044	0.047	0.350	0.035
		8.97–14.14	48 (24.7%)	12 (25.5%)	13 (26.0%)				
		14.18–20.52	54 (27.8%)	6 (12.8%)	12 (24.0%)				
		21.46–68.93	54 (27.8%)	12 (25.5%)	7 (14.0%)				
	OS-Based Optimization	1.79–7.15	19 (9.8%)	13 (27.7%)	7 (14.0%)	0.002 (0.007)	0.390 (0.780)	0.100 (0.120)	0.005 (0.018)
		**7.17–68.93**⁎	175 (90.2%)	34 (72.3%)	43 (86.0%)				
	DFS-Based Optimization	**1.79–17.91**⁎	124 (63.9%)	33 (70.2%)	39 (78.0%)	0.420 (0.560)	0.060 (0.210)	0.380 (0.350)	0.150 (0.340)
		18.21–68.93	70 (36.1%)	14 (29.8%)	11 (22.0%)				
									
Leptin (ng/ml)	Median (25–75th)	–	26.0 (16.9–38.0)	23.0 (15.4–44.1)	32.0 (21.8–50.1)	0.820 (0.330)	0.050 (0.120)	0.210 (0.700)	0.150 (0.160)
	Quartiles	BLQ to 17.00	50 (25.8%)	15 (31.9%)	8 (16.0%)	0.180	0.250	0.180	0.190
		17.73–27.07	49 (25.3%)	12 (25.5%)	12 (24.0%)				
		27.09–41.75	53 (27.3%)	6 (12.8%)	13 (26.0%)				
		43.06–159.15	42 (21.6%)	14 (29.8%)	17 (34.0%)				
	OS-Based Optimization	**BLQ to 6.17**⁎	14 (7.2%)	3 (6.4%)	1 (2.0%)	1.000 (0.640)	0.320 (0.890)	0.350 (0.740)	0.450 (0.850)
		6.25–159.15	180 (92.8%)	44 (93.6%)	49 (98.0%)				
	DFS-Based Optimization	BLQ to 50.82	155 (79.9%)	37 (79.9%)	39 (78.0%)	0.860 (0.070)	0.770 (0.002)	0.930 (0.070)	0.950 (0.002)
		**51.64–159.15**⁎	39 (20.1%)	10 (20.1%)	11 (22.0%)				
									
CRP (μg/ml)	Median (25–75th)	–	2.10 (0.80–4.65)	2.80 (1.10–5.30)	3.05 (1.30–9.15)	0.340 (0.670)	0.022 (0.370)	0.260 (0.890)	0.060 (0.750)
	Quartiles	BLQ to 0.90	56 (28.9%)	9 (19.1%)	9 (18.0%)	0.490	0.160	0.770	0.340
		1.00–2.20	47 (24.2%)	14 (29.8%)	11 (22.0%)				
		2.30–5.20	49 (25.3%)	11 (23.4%)	12 (24.0%)				
		5.30–23.00	42 (21.6%)	13 (27.7%)	18 (36.0%)				
	OS-Based Optimization	BLQ to 8.30	173 (89.2%)	41 (87.2%)	34 (68.0%)	0.001 (0.390)	0.710 (0.580)	0.028 (0.250)	0.001 (0.530)
		**8.60–23.00**⁎	21 (10.8%)	6 (12.8%)	16 (32.0%)				
	DFS-Based Optimization	BLQ to 16.60	186 (95.9%)	46(97.9%)	45 (90.0%)	1.000 (0.300)	0.150 (0.670)	0.210 (0.180)	0.190 (0.470)
		**17.20–23.00**	8 (4.1%)	1 (2.1%)	5 (10.0%)				
									
IL-6 (pg/ml)	Median (25–75th)	–	0.7 (0.44–1.76)	1.49 (0.59–3.72)	1.14 (0.51–3.10)	0.010 (0.090)	0.170 (0.740)	0.330 (0.048)	0.024 (0.180)
	Quartiles	BLQ to 0.44	55 (28.4%)	7 (14.9%)	12 (24.0%)	0.027	0.190	0.670	0.060
		0.50–0.70	58 (29.9%)	9 (19.1%)	9 (18.0%)				
		0.72–2.32	39 (20.1%)	16 (34.0%)	13 (26.0%)				
		2.51–138.00	42 (21.6%)	15 (31.9%)	16 (32.0%)				
	OS-Based Optimization	**BLQ**⁎	18 (9.3%)	0 (0.0%)	1 (2.0%)	0.028 (0.010)	1.000 (0.999)	0.140 (0.300)	0.022 (0.031)
		0.34–138.00	176 (90.7%)	47 (100%)	49 (98.0%)				
	DFS-Based Optimization	**BLQ**⁎	18 (9.3%)	0 (0.0%)	1 (2.0%)	0.028 (0.010)	1.000 (0.999)	0.140 (0.300)	0.022 (0.031)
		0.34–138.00	176 (90.7%)	47 (100%)	49 (98.0%)				
									
TNF-α (pg/ml)	Median (25–75th)	–	5.55 (3.86–8.22)	6.64 (4.41–11.41)	6.53 (4.89–9.20)	0.060 (0.080)	0.080 (0.420)	0.850 (0.300)	0.070 (0.170)
	Quartiles	BLQ to 4.19	56 (28.9%)	9 (19.1%)	8 (16.0%)	0.060	0.260	0.480	0.120
		4.21–5.66	46 (23.7%)	14 (29.8%)	13 (26.0%)				
		5.67–8.73	51 (26.3%)	7 (14.9%)	14 (28.0%)				
		8.90–77.00	41 (21.1%)	17 (36.2%)	15 (30.0%)				
	OS-Based Optimization	BLQ to 8.96	153 (78.9%)	31 (66.0%)	36 (72.0%)	0.060 (0.150)	0.300 (0.390)	0.520 (0.650)	0.150 (0.320)
		**9.00–77.00**⁎	41 (21.1%)	16 (34.0%)	14 (28.0%)				
	DFS-Based Optimization	BLQ to 8.96	153 (78.9%)	31 (66.0%)	36 (72.0%)	0.060 (0.150)	0.300 0.390)	0.520 (0.650)	0.150 (0.320)
		**9.00–77.00**⁎	41 (21.1%)	16 (34.0%)	14 (28.0%)				
									
IL-1β (pg/ml)	Median (25–75th)	–	1.60 (1.60–3.20)	1.60 (1.60–3.75)	1.60 (1.60–2.76)	0.170 (0.030)	0.140 (0.250)	0.037 (0.020)	0. 090 (0.011)
	OS-Based Optimization	**BLQ to 13.08**⁎	187 (96.4%)	40 (85.1%)	50 (100%)	0.008 (0.007)	0.350 (0.035)	0.005 (0.001)	0.002 (0.001)
		14.74–127.08	7 (3.6%)	7 (14.9%)	0 (0.0%)				
	DFS-Based Optimization	**BLQ to 13.08**⁎	187 (96.4%)	40 (85.1%)	50 (100%)	0.008 (0.007)	0.350 (0.035)	0.005 (0.001)	0.002 (0.001)
		14.74–127.08	7 (3.6%)	7 (14.9%)	0 (0.0%)				
									
C-peptide (ng/ml)	Median (25–75th)	–	1.67 (1.17–2.42)	2.36 (1.33–3.20)	2.26 (1.84–3.14	0.050 (0.760)	<0.001 (0.041)	0.330 (0.060)	<0.001 (0.090)
	Quartiles	0.14–1.28	58 (29.9%)	11 (23.4%)	4 (8.0%)	0.043	<0.001	0.140	<0.001
		1.29–1.82	59 (30.4%)	7 (14.9%)	7 (14.0%)				
		1.83–2.68	37 (19.1%)	13 (27.7%)	22 (44.0%)				
		2.68–9.02	40 (20.6%)	16 (34.0%)	17 (34.0%)				
	OS-Based Optimization	**0.14–0.75**⁎	14 (7.2%)	7 (14.9%)	0 (0%)	0.14 (0.037)	0.080 (0.130)	0.005 (0.001)	0.013 (0.008)
		0.76–9.02	180 (92.8%)	40 (85.1%)	50 (100%)				
	DFS-Based Optimization	**0.14–0.75**⁎	14 (7.2%)	7 (14.9%)	0 (0%)	0.140 (0.037)	0.080 (0.130)	0.005 (0.001)	0.013 (0.008)
		0.76–9.02	180 (92.8%)	40 (85.1%)	50 (100%)				

C-reactive protein (CRP), interleukine-6 (IL-6), interleukine-1β (IL-1β), interleukine-1Ra (IL-1Ra), tumor necrosis factor-α (TNF-α).

⁎ Overall survival (OS)– and disease-free survival (DFS)-optimized biomarker ranges associated with poorer outcomes are represented in bold. BLQ=below limit of quantitation. MVP=*p*-value of the multivariate adjusted analysis.

**Table 2:** Adipokine correlations and secretagogue use.

Compared biomarkers		Group	Unadjusted correlation			Adjusted correlation			
			Pearson correlation	95% CI	*p*-value	Pearson correlation		95% CI	*p*-value
C-peptide	IL-1β	All Subjects (*n*=291)	−0.089	−0.202 to 0.027	0.132	−0.081		−0.194 to 0.034	0.168
		Controls (*n*=194)	−0.003	−0.145 to 0.139	0.967	0.01		−0.131 to 0.151	0.891
		No Secretagogue (*n*=43)	−0.265	−0.532 to 0.051	0.095	−0.285		−0.539 to 0.017	0.061
		Any Secretagogue (*n*=54)	−0.069	−0.338 to 0.211	0.63	−0.105		−0.363 to 0.167	0.446
								
C-peptide	IL-1Ra	All Subjects (*n*=291)	−0.081	−0.195 to 0.034	0.167	−0.073		−0.187 to 0.042	0.212
		Controls (*n*=194)	−0.075	−0.214 to 0.068	0.304	−0.063		−0.202 to 0.079	0.382
		No Secretagogue (*n*=43)	−0.171	−0.458 to 0.148	0.287	−0.18		−0.455 to 0.128	0.245
		Any Secretagogue (*n*=54)	0.064	−0.215 to 0.334	0.653	0.004		−0.264 to 0.272	0.977
								
C-peptide	IL-6	All Subjects (*n*=291)	−0.053	−0.168 to 0.063	0.368	−0.068		−0.182 to 0.047	0.244
		Controls (*n*=194)	−0.046	−0.187 to 0.097	0.528	−0.059		−0.198 to 0.083	0.414
		No Secretagogue (*n*=43)	−0.146	−0.437 to 0.174	0.366	−0.159		−0.438 to 0.149	0.306
		Any Secretagogue (*n*=54)	−0.022	−0.295 to 0.255	0.879	0.032		−0.238 to 0.297	0.819
								
C-peptide	Adiponectin	All Subjects (*n*=291)	**−0.163**	**−0.274 to −0.048**	**0.005**	**−0.178**		**−0.287 to −0.064**	**0.002**
		Controls (*n*=194)	**−0.145**	**−0.281 to −0.003**	**0.045**	−0.119		−0.255 to 0.022	0.098
		No Secretagogue (*n*=43)	**−0.343**	**−0.591 to −0.035**	**0.028**	**−0.388**		**−0.617 to −0.1**	**0.009**
		Any Secretagogue (*n*=54)	−0.086	−0.353 to 0.194	0.547	−0.068		−0.33 to 0.203	0.621
								
C-peptide	Leptin	All Subjects (*n*=291)	**0.161**	**0.047 to 0.272**	**0.006**	**0.238**		**0.126 to 0.343**	**<0.001**
		Controls (*n*=194)	**0.278**	**0.141 to 0.404**	**<0.001**	**0.314**		**0.181 to 0.436**	**<0.001**
		No Secretagogue (*n*=43)	−0.042	−0.349 to 0.273	0.795	−0.001		−0.301 to 0.299	0.995
		Any Secretagogue (*n*=54)	0.03	−0.248 to 0.303	0.834	0.144		−0.129 to 0.396	0.297
								
C-peptide	CRP	All Subjects (*n*=291)	−0.075	−0.188 to 0.041	0.207	0.023		−0.092 to 0.137	0.698
		Controls (*n*=194)	−0.117	−0.254 to 0.026	0.107	−0.042		−0.182 to 0.099	0.556
		No Secretagogue (*n*=43)	0.192	−0.127 to 0.475	0.231	0.207		−0.099 to 0.478	0.179
		Any Secretagogue (*n*=54)	−0.086	−0.353 to 0.194	0.545	–0.014		−0.281 to 0.255	0.92
								
C-peptide	TNFα	All Subjects (*n*=291)	−0.012	−0.127 to 0.104	0.839	0.035		−0.08 to 0.15	0.55
		Controls (*n*=194)	0.086	−0.056 to 0.226	0.234	0.125		−0.016 to 0.261	0.082
		No Secretagogue (*n*=43)	−0.3	−0.559 to 0.013	0.057	−0.277		−0.533 to 0.026	0.069
		Any Secretagogue (*n*=54)	0.265	−0.011 to 0.504	0.057	0.227		−0.043 to 0.467	0.096
IL-1β	IL-1Ra	All Subjects (*n*=291)	**0.753**	**0.698 to 0.799**	**<0.001**	**0.75**		**0.695 to 0.797**	**<0.001**
		Controls (*n*=194)	**0.436**	**0.313 to 0.544**	**<0.001**	**0.435**		**0.313 to 0.542**	**<0.001**
		No Secretagogue (*n*=43)	**0.932**	**0.874 to 0.964**	**<0.001**	**0.929**		**0.871 to 0.961**	**<0.001**
		Any Secretagogue (*n*=54)	**0.367**	**0.101 to 0.583**	**0.007**	**0.384**		**0.13 to 0.591**	**0.004**
								
IL-1β	IL-6	All Subjects (*n*=291)	**0.339**	**0.232 to 0.437**	**<0.001**	**0.337**		**0.231 to 0.435**	**<0.001**
		Controls (*n*=194)	**0.484**	**0.367 to 0.586**	**<0.001**	**0.476**		**0.36 to 0.578**	**<0.001**
		No Secretagogue (*n*=43)	**0.69**	**0.482 to 0.824**	**<0.001**	**0.682**		**0.481 to 0.816**	**<0.001**
		Any Secretagogue (*n*=54)	0.042	−0.237 to 0.314	0.771	0.055		−0.216 to 0.318	0.694
								
IL-1β	Adiponectin	All Subjects (*n*=291)	−0.038	−0.153 to 0.077	0.515	−0.024		−0.138 to 0.091	0.685
		Controls (*n*=194)	−0.055	−0.195 to 0.088	0.451	−0.031		−0.171 to 0.11	0.665
		No Secretagogue (*n*=43)	−0.047	−0.353 to 0.269	0.773	−0.001		−0.301 to 0.3	0.996
		Any Secretagogue (*n*=54)	−0.033	−0.306 to 0.245	0.818	−0.054		−0.317 to 0.217	0.695
								
IL-1β	Leptin	All Subjects (*n*=291)	0	−0.116 to 0.115	0.994	−0.009		−0.124 to 0.106	0.88
		Controls (*n*=194)	0.072	−0.071 to 0.212	0.322	0.081		−0.06 to 0.22	0.259
		No Secretagogue (*n*=43)	−0.045	−0.351 to 0.27	0.782	−0.092		−0.382 to 0.214	0.553
		Any Secretagogue (*n*=54)	−0.046	−0.317 to 0.233	0.749	−0.202		−0.446 to 0.069	0.14
								
IL-1β	CRP	All Subjects (*n*=291)	−0.023	−0.139 to 0.092	0.693	−0.029		−0.143 to 0.086	0.623
		Controls (*n*=194)	−0.019	−0.16 to 0.124	0.799	−0.01		−0.151 to 0.131	0.891
		No Secretagogue (*n*=43)	0.038	−0.276 to 0.346	0.813	−0.009		−0.309 to 0.292	0.953
		Any Secretagogue (*n*=54)	−0.05	−0.322 to 0.228	0.724	−0.14		−0.393 to 0.133	0.31
								
IL-1β	TNFα	All Subjects (*n*=291)	**0.487**	**0.394 to 0.571**	**<0.001**	**0.484**		**0.391 to 0.568**	**<0.001**
		Controls (*n*=194)	**0.196**	**0.055 to 0.328**	**0.007**	**0.208**		**0.069 to 0.339**	**0.004**
		No Secretagogue (*n*=43)	**0.668**	**0.45 to 0.811**	**<0.001**	**0.618**		**0.39 to 0.775**	**<0.001**
		Any Secretagogue (*n*=54)	−0.065	−0.334 to 0.215	0.651	−0.007		−0.274 to 0.261	0.961
								
IL-1Ra	IL-6	All Subjects (*n*=291)	**0.338**	**0.231 to 0.436**	**<0.001**	**0.335**		**0.229 to 0.433**	**<0.001**
		Controls (*n*=194)	**0.319**	**0.186 to 0.441**	**<0.001**	**0.31**		**0.177 to 0.432**	**<0.001**
		No Secretagogue (*n*=43)	**0.759**	**0.587 to 0.866**	**<0.001**	**0.748**		**0.578 to 0.856**	**<0.001**
		Any Secretagogue (*n*=54)	0.021	−0.256 to 0.295	0.882	−0.029		−0.294 to 0.241	0.836
IL-1Ra	Adiponectin	All Subjects (*n*=291)	−0.043	−0.158 to 0.073	0.467	−0.049		−0.163 to 0.067	0.407
		Controls (*n*=194)	−0.013	−0.155 to 0.129	0.859	−0.033		−0.173 to 0.108	0.643
		No Secretagogue (*n*=43)	−0.077	−0.379 to 0.241	0.637	−0.064		−0.358 to 0.241	0.68
		Any Secretagogue (*n*=54)	−0.105	−0.37 to 0.175	0.46	−0.147		−0.399 to 0.126	0.287
								
IL-1Ra	Leptin	All Subjects (*n*=291)	0.021	−0.095 to 0.136	0.727	0.028		−0.087 to 0.143	0.63
		Controls (*n*=194)	0.017	−0.125 to 0.159	0.812	0.055		−0.087 to 0.194	0.447
		No Secretagogue (*n*=43)	0.046	−0.269 to 0.353	0.774	0.004		−0.296 to 0.304	0.977
		Any Secretagogue (*n*=54)	−0.101	−0.366 to 0.18	0.478	-0.131		−0.385 to 0.142	0.344
								
IL-1Ra	CRP	All Subjects (*n*=291)	0.066	−0.05 to 0.18	0.263	0.071		−0.045 to 0.184	0.229
		Controls (*n*=194)	**0.147**	**0.005 to 0.283**	**0.042**	**0.166**		**0.026 to 0.3**	**0.02**
		No Secretagogue (*n*=43)	0.058	−0.259 to 0.363	0.722	0.042		−0.262 to b0.338	0.79
		Any Secretagogue (*n*=54)	−0.081	−0.349 to 0.199	0.569	−0.1		−0.358 to 0.172	0.47
								
IL-1Ra	TNFα	All Subjects (*n*=291)	**0.529**	**0.441 to 0.608**	**<0.001**	**0.516**		**0.426 to 0.596**	**<0.001**
		Controls (*n*=194)	**0.456**	**0.336 to 0.562**	**<0.001**	**0.449**		**0.329 to 0.555**	**<0.001**
		No Secretagogue (*n*=43)	**0.623**	**0.386 to 0.782**	**<0.001**	**0.578**		**0.335 to 0.748**	**<0.001**
		Any Secretagogue (*n*=54)	0.202	−0.078 to 0.452	0.152	0.203		−0.068 to 0.447	0.138
								
IL-6	Adiponectin	All Subjects (*n*=291)	−0.062	−0.176 to 0.054	0.294	−0.05		−0.164 to 0.066	0.398
		Controls (*n*=194)	−0.103	−0.242 to 0.039	0.155	−0.088		−0.226 to 0.054	0.222
		No Secretagogue (*n*=43)	0.076	−0.242 to 0.378	0.64	0.112		−0.195 to 0.399	0.472
		Any Secretagogue (*n*=54)	−0.07	−0.339 to 0.209	0.623	−0.043		−0.307 to 0.228	0.759
								
IL-6	Leptin	All Subjects (*n*=291)	0.055	−0.061 to 0.169	0.354	0.015		−0.101 to 0.129	0.804
		Controls (*n*=194)	0.054	−0.089 to 0.195	0.457	0.01		−0.131 to 0.151	0.888
		No Secretagogue (*n*=43)	0.069	−0.248 to 0.372	0.672	0.081		−0.225 to 0.372	0.603
		Any Secretagogue (*n*=54)	0.104	−0.176 to 0.369	0.464	0.081		−0.191 to 0.341	0.559
								
IL-6	CRP	All Subjects (*n*=291)	0.096	−0.02 to 0.209	0.104	0.059		−0.056 to 0.173	0.315
		Controls (*n*=194)	0.141	−0.001 to 0.277	0.051	0.095		−0.047 to 0.233	0.188
		No Secretagogue (*n*=43)	−0.093	−0.394 to 0.225	0.564	−0.09		−0.38 to 0.216	0.562
		Any Secretagogue (*n*=54)	**0.302**	**0.028 to 0.533**	**0.029**	**0.268**		**0.001 to 0.5**	**0.047**
IL-6	TNFα	All Subjects (*n*=291)	**0.243**	**0.131 to 0.349**	**<0.001**	**0.224**		**0.112 to 0.33**	**<0.001**
		Controls (*n*=194)	**0.262**	**0.124 to 0.389**	**<0.001**	**0.24**		**0.102 to 0.368**	**0.001**
		No Secretagogue (*n*=43)	**0.43**	**0.137 to 0.654**	**0.005**	**0.437**		**0.157 to 0.652**	**0.003**
		Any Secretagogue (*n*=54)	**0.309**	**0.036 to 0.539**	**0.026**	**0.304**		**0.039 to 0.528**	**0.024**
Adiponectin	Leptin	All Subjects (*n*=291)	−0.085	−0.198 to 0.031	0.152	**−0.15**		**−0.261 to −0.036**	**0.01**
		Controls (*n*=194)	**−0.235**	**−0.365 to −0.096**	**0.001**	**−0.262**		**−0.389 to −0.126**	**<0.001**
		No Secretagogue (*n*=43)	0.09	−0.228 to 0.391	0.577	0.003		−0.298 to 0.303	0.986
		Any Secretagogue (*n*=54)	**0.392**	**0.131 to 0.603**	**0.004**	**0.278**		**0.011 to 0.508**	**0.04**
								
Adiponectin	CRP	All Subjects (*n*=291)	−0.105	−0.218 to 0.01	0.073	**−0.185**		**−0.294 to −0.072**	**0.002**
		Controls (*n*=194)	−0.013	−0.154 to 0.13	0.861	−0.099		−0.237 to 0.043	0.169
		No Secretagogue (*n*=43)	−0.222	−0.499 to 0.097	0.165	**−0.299**		**−0.55 to 0.002**	**0.049**
		Any Secretagogue (*n*=54)	**−0.32**	**−0.547 to −0.049**	**0.02**	**−0.309**		**−0.533 to −0.045**	**0.021**
								
Adiponectin	TNFα	All Subjects (*n*=291)	−0.032	−0.147 to 0.084	0.589	−0.009		−0.124 to 0.106	0.874
		Controls (*n*=194)	−0.031	−0.172 to 0.112	0.671	0.011		−0.13 to 0.152	0.874
		No Secretagogue (*n*=43)	−0.025	−0.334 to 0.289	0.878	0.019		−0.283 to 0.318	0.902
		Any Secretagogue (*n*=54)	−0.037	−0.309 to 0.241	0.795	−0.031		−0.296 to 0.239	0.825
								
Leptin	CRP	All Subjects (*n*=291)	−0.103	−0.216 to 0.013	0.08	0.114		−0.001 to 0.226	0.051
		Controls (*n*=194)	**−0.151**	**−0.287 to −0.009**	**0.036**	0.07		−0.072 to 0.208	0.334
		No Secretagogue (*n*=43)	−0.141	−0.433 to 0.178	0.382	0.165		−0.142 to 0.443	0.286
		Any Secretagogue (*n*=54)	−0.052	−0.323 to 0.227	0.714	0.173		−0.099 to 0.421	0.208
								
Leptin	TNFα	All Subjects (*n*=291)	0.087	−0.029 to 0.2	0.142	**0.127**		**0.012 to 0.238**	**0.03**
		Controls (*n*=194)	0.03	−0.112 to 0.171	0.679	0.094		−0.048 to 0.231	0.193
		No Secretagogue (*n*=43)	0.082	−0.236 to 0.384	0.613	0.208		−0.099 to 0.478	0.178
		Any Secretagogue (*n*=54)	0.214	−0.065 to 0.463	0.128	0.068		−0.203 to 0.33	0.623
								
TNFα	CRP	All Subjects (*n*=291)	0.021	−0.095 to 0.136	0.721	0.056		−0.059 to 0.17	0.337
		Controls (*n*=194)	0.101	−0.042 to 0.24	0.164	0.136		−0.005 to 0.271	0.058
		No Secretagogue (*n*=43)	0.032	−0.282 to 0.34	0.843	0.072		−0.233 to 0.365	0.644
		Any Secretagogue (*n*=54)	−0.076	−0.344 to 0.204	0.595	−0.126		−0.381 to 0.147	0.361

Significant correlations are displayed in bolded text. The differences that are only significant in either adjusted or unadjusted correlations are further denoted by an outline. C-reactive protein (CRP), interleukine-6 (IL-6), interleukine-1β (IL-1β), interleukine-1Ra (IL-1Ra), tumor necrosis factor-α (TNF-α), confidence interval (CI).
